# Reprocessing Zamak laryngoscope blades into new instrument parts; an ‘all-in-one’ experimental study

**DOI:** 10.1016/j.heliyon.2022.e11711

**Published:** 2022-11-17

**Authors:** Bart van Straten, Brian Tantuo, Jenny Dankelman, Nicolaas H. Sperna Weiland, Bendiks Jan Boersma, Tim Horeman

**Affiliations:** a Department of BioMechanical Engineering, Faculty of Mechanical, Maritime and Materials Engineering, Delft University of Technology, Delft, the Netherlands; b Department of Process & Energy, Faculty of Mechanical, Maritime and Materials Engineering, Delft University of Technology, Delft, the Netherlands; c Department of Anaesthesiology and Centre for Sustainable Healthcare, Amsterdam University Medical Center, Amsterdam, the Netherlands

**Keywords:** Circular economy, Recycle, Sustainable production and consumption, Cleaner production, Sustainable business, Clean technology

## Abstract

**Introduction:**

Disposable instruments in healthcare have led to a significant increase of medical waste. The aim of this study is to validate the recycling of disposable Zamak laryngoscope blades into new medical components by using a new ‘all-in-one’ affordable reprocessing setup as alternative for die-casting.

**Methods:**

A n “all-in-one” casting set-up was designed and built. Laryngoscope blades, recovered from two hospitals, were disinfected, melted and cast into dog-bones and into new instrument parts. The quality of the cast material was evaluated using X-ray fluorescence spectrometry. The mechanical properties were obtained by assessing the Ultimate Tensile Strength (UTS) and tensile tests.

**Results:**

A recovery of 93 % Zamak was obtained using a melting temperature of 420 °C for 3 h. The XRF Spectro data showed higher Zinc and silicon concentrations when compared with Virgin Zamak. The dog-bones tests resulted in an average UTS, Yield Strength (YS) and Young's Modulus (YM) of 236 ± 61 (MPa), 70 ± 43 and 9 ± 3, respectively, representing 82 %, 103 % and 64 % of the UTS, YS and YM of standard Zamak. Functional instrument parts with extensions and inner chambers were cast with a maximal shrinkage percentage of 1 ± 1 %.

**Discussion:**

This study demonstrates that the created “all-in-one” reprocessing method can process contaminated disposable Zamak laryngoscope blades into new raw base material and new instrument parts. Although material and surface properties can deteriorate, reprocessed Zamak still has sufficient mechanical properties and can be used to cast complex parts with sufficient dimensional tolerances and minimal shrinkage.

**Conclusion:**

A micro reprocessing method was designed and used to turn disposed laryngoscope blades into new basis material and semi-finished components. Follow up studies are needed to scale and optimize this process towards a functional alternative for die casting. It should be further investigated how this process can contribute to further medical waste reduction and a circular healthcare economy.

## 1 Introduction

Health care waste has been growing significantly as a result of the growing population and the use of disposable products [1, 2]. Operating rooms are among the largest contributors of medical waste produced in hospitals as a result of disposable surgical supplies [3] creating huge amounts of waste [4].

In line with the circular economy (CE) philosophy, waste should be minimized and seen as a valuable input material for the manufacturing of raw base materials for new products. Waste can be prevented by conducting proper maintenance, repair or refurbishment to extend the product's life cycle. Recycling is preferred when the previous is not possible [5]. By reusing medical waste, the valuable materials are preserved, greenhouse gas (GHG) emissions are lowered and the environmental impact is reduced [1, 6, 7].

The European Green Deal stimulates sectors to participate in achieving a circular economy [8]. The aim is to become climate neutral by 2050 [9,10]. Healthcare waste in high income countries vary between 1.7 kg and 8.4 kg per bed per day [11]. Other countries vary from 6.7–7.9 kg [12]. Hospitals in the Netherlands are responsible for around 7 % of the total carbon footprint [13] meaning that improvements can be made with regard to waste processing and CO_2_ emissions.

**Figure 1:**
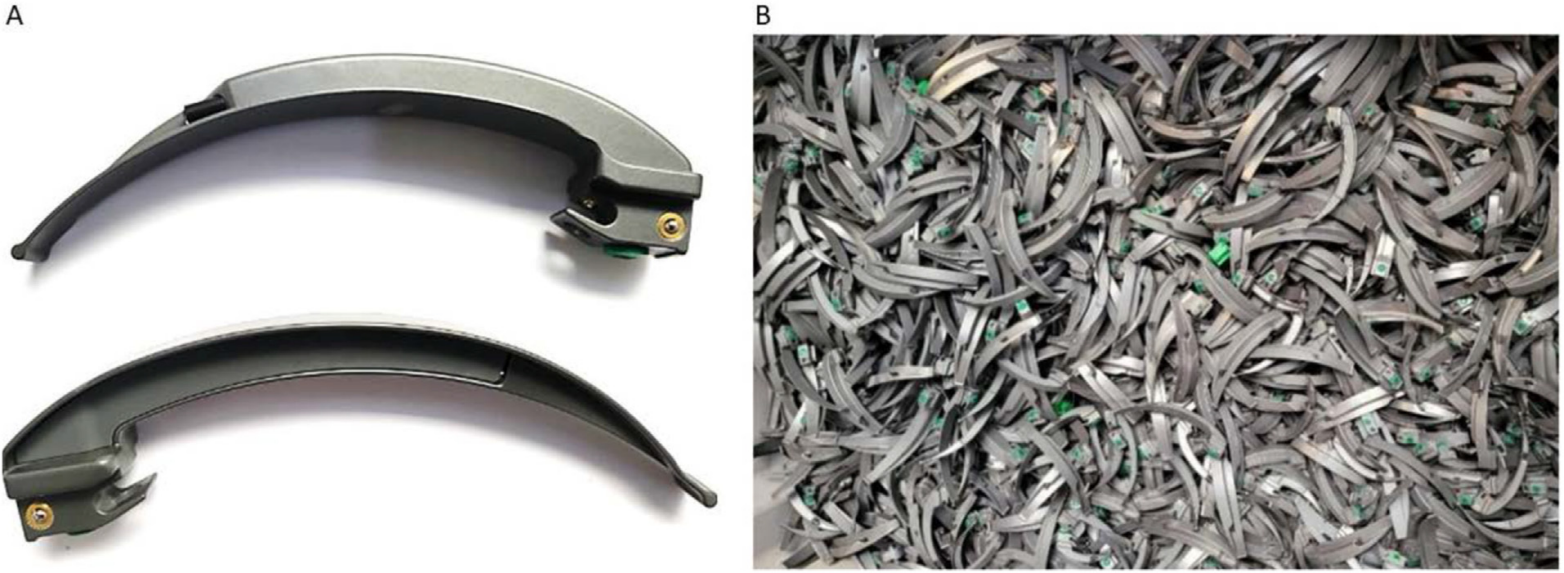
A: Zamak Laryngoscope blades. Left, the Mackintosh type Laryngoscope Blade. B: Right, Laryngoscope blades disposed after use.

An analysis of the environmental impact when using disposable metal instruments indicated that disposable stainless-steel scissors had the highest negative environmental impact and appeared to have higher total cost of ownership than reusable scissors [14]. In line with these findings it was of interest to investigate other metal disposable instruments used in high quantities in the Operating Room (OR). During endotracheal intubation, laryngoscope blades are used in large quantities every day as either a disposable or reusable product. Due to the combination of the complex shaped blade with disposable parts such as an optical fibers and locking members, cleaning becomes difficult and sterilization costs are increased. Driven by concerns regarding the prion transferable NvCJD in the past [15], there was a trend towards the manufacturing of the disposable versions of these blades. Disposable metal blades such as the laryngoscope blades from Teleflex are made from a non-ferrous metal alloy named Zamak. Zamak allows for rapid casting of disposable complex shapes [16]. Figure 1 shows an image of the disposable Rüsch Polaris Fiber Optic Laryngoscope Blade (Rüsch Specialty Products, Teleflex, Dublin Road Westmeath, Ireland). Laryngoscope blades are subjected to certification according to existing standard ISO 7376 where the blade must meet minimum strength properties and be resistant to deformability Laryngoscope blades are subject to certification according to existing standard ISO 7376. When considering the development of new medical products that undergo high deformation and or loading, is important to check both the Ultimate Strength and deformability of the cast material.

### 1.1 Zamak laryngoscope blades

Zamak consists of Zinc, Aluminum, Magnesium and Copper [17]. The laryngoscope blades as shown in Figure 1 are disposed after use on the OR. Zamak 3 is the most common type of material and used for casting of medical components. The standard composition is 3.5–4.3 % aluminum, 0.02–0.06 % magnesium, a maximum of 0.25 % copper with the remainder being zinc (≈95 %) [18]. The function of aluminum and copper is to increase the strength. The function of magnesium is to prevent corrosion [19]. Table 1 shows the standard and mechanical properties of Zamak 3. The main body of the Teleflex laryngoscope blade is made from Zamak that is covered by an epoxy-polyester coating and contains three inserts with a diameter of 4 mm made of both stainless steel and brass. The influence of metal impurities and presence of other alloys may influence the occurrence of defects and diminish material properties [20, 21] after recasting. To ensure a high quality end-product, the influence of the melting, casting and presence of undesired plastics or metals should be investigated.

**Table 1:** Standard thermal and mechanical properties of Zamak 3.

Zamak 3 [11]	Value
Melting Temperature – Liquidus (Celsius)	390 °C
Melting Temperature – Solidus (Celsius)	380 °C
Viscosity [12] (Pa s)	≈3.5 mPa∗s @ 400 °C
Solidification shrinkage (%)	1.2 %
Ultimate Tensile Strength (Mpa)	280 MPa
Yield strength (0.2% offset)	210 MPa
Young's modulus	86 GPa
Elongation at Break	11 %

The casting of Zamak is commonly done through die-casting, a metal casting process where molten metal is forced under high pressure through a system into a mold [22]. This specific casting process is often used to produce geometrically complex shaped metal parts and requires expensive die-casting machines and processes [23]. Besides the need for dedicated machinery, the die-casting process involves critical optimization of injection parameters and mold configurations to prevent that gas is entrapped in the cast resulting in the formation of pores in the cast material [24]. Therefore, a need exists to develop an alternative and a less complex ‘all-in-one’ set-up in which disposed medical instruments can be disinfected, melted and directly molded into new end-products.

### 1.2 Aim

The aim of this study was to validate the recycling of disposable Zamak laryngoscope blades into new medical components by using a new ‘all-in-one’ affordable reprocessing setup as alternative for die-casting.

## 2 Methods

Two batches of laryngoscope blades were collected from the Spaarne Hospital (Hoofddorp, the Netherlands) and the Amsterdam University Medical Center (Amsterdam, the Netherlands) and melted and cast. The OR staff deposited the waste in special containers with a lockable lid. The containers were opened after receipt, disinfected at 90 °C and the blades were put in a larger collection bin until they were further processed. A process diagram of the different instrument flows during reprocessing is added in Supplemental file 1*.*

**Figure 2:**
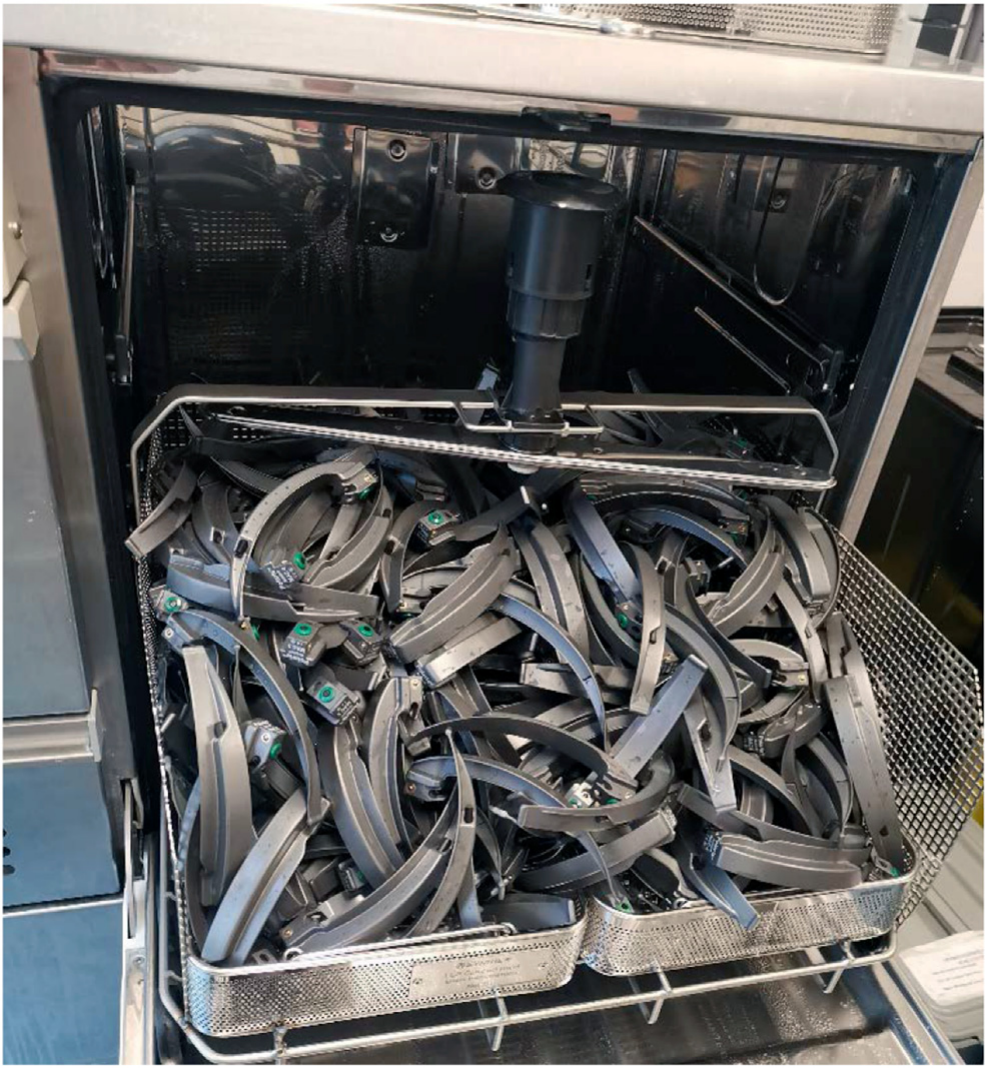
Thermal disinfection with customized insert rack for disinfection of blades in larger quantities.

Figure 2 shows how the blades were disinfected in a modified G7782 CD Miele medical thermo disinfector at 90 °C (Miele Nederland, Vianen, the Netherlands).

**Figure 3:**
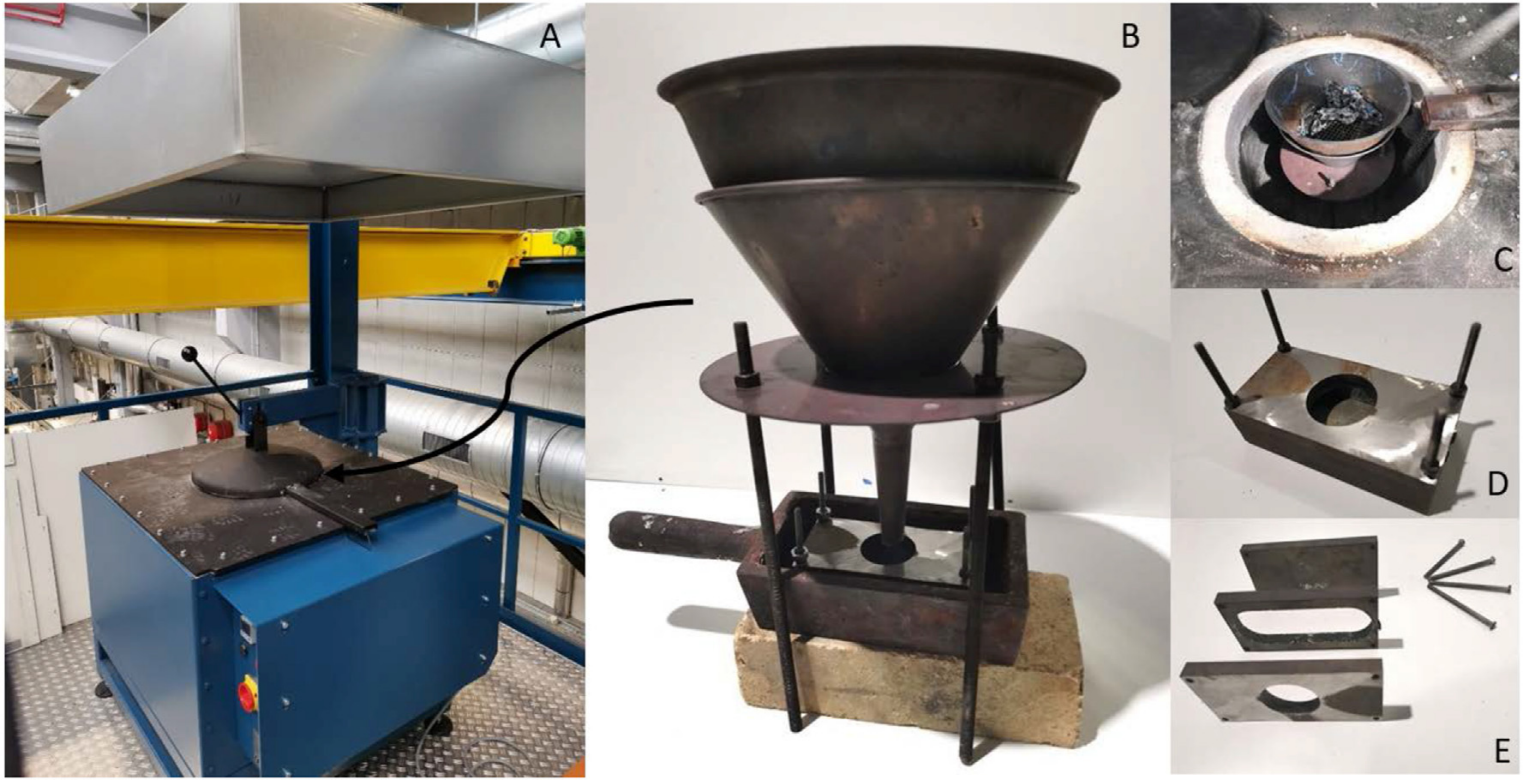
Melting Oven. A: The induction furnace for melting of the Zamak laryngoscope blades. B: All-in-one casting setup placed in the melting oven. C: Top bowl with filter allows only the Zamak to pass. D: Cast with riser. E: Exploded view cast with riser.

The blades were manually put in the stainless steel bowl and funnel and placed in the melting oven. The blades were melted into ingots and cleaned ultrasonically. The melting and casting was done in a single production line, based on a single location at the Sustainable Surgery Lab of Delft University of Technology (Supplemental file 2). An induction furnace (electric melting oven, KOS, series 219029) was used for melting the laryngoscope blades (Figure 3). The melting oven contains a cylindrical crucible with a diameter of 395 mm and a height of 345 mm as maximal space for the “all-in-one” melting process consisting of an induction furnace, a casting setup with a bowl with filter and casting mold with a riser (Figure 3A–E).

After melting, the flow of the liquid Zamak was propagated into a mold by gravity casting. The adhesion of the epoxy-polyester coating is reduced with the bake-off method [25]. Earlier studies show that the bake-off requires heating of a coated item to 340 °C–400 °C to turn the coating starts turning to ashes while keeping the inserts made from stainless steel and brass intact. At these temperatures, the coating starts flaking and forming cracks. Once the coating has been degraded enough, the Zamak flows into the mold. This process usually takes between three to 6 h. During oxidation, gases and debris can be absorbed by the Zamak, while in its liquid form it may create defects in the form of porosity during casting. To reduce the oxidation rate, a melting temperature of 420 °C was chosen for the experiments as this lies within the margin of the Zamak recommended melting temperature (395 °C and 425 °C) [26]. The used Zamak blades also contains a tube made of a shielded transparent fiber held in place by a polyvinylchloride part.

### 2.1 Melting setup

The melting setup consisted of a flat bottom stainless steel bowl with a grate on the bottom and a stainless-steel ingot mold (Figure 3). The grate acted as a filter to prevent the coating residue, inserts and the particles from plastic parts to flow along into the mold. The funnel was held up by a stand and had the 3 × 3 mm hole grate. The mold was placed below the bottom funnel hole. The conical shape of the funnel was used to have all of the liquid converging towards the funnel opening supported by gravity, contributing to a higher Zamak recovery.

During solidification, the melted material decreases its volume and the casting shrinks. The particles are pulled in the direction where the solidification starts, tending to start at the walls of the mold. This causes cavities to appear if not enough melt is available to compensate for the lost volume named shrinkage porosity [27]. To prevent this shrinkage porosity, the ingot mold was designed with a riser, a reservoir for casting. The melted material in the riser solidifies last. Therefore, the mold can pull from the riser towards areas where cavities would otherwise emerge. During the design of the riser volume and opening diameter, Chvorinov's rule in relation to the solidification time ratio between riser and mold was taken into account stating that the solidification time (1) of a casting depends on the relation between the volume and surface area according to [28]:

(1) $$t={B\;\left(\frac{V}{A}\right)}^{2}$$

t = Solidification time in [s]

B = Mold constant in [s·m^−2^]

V = Volume in [m^3^]

A = Surface area in [m^2^]

According to DeGarmo [29], a 25% difference between the solidification time of the casting and the riser is sufficient. Because the riser and the rest of the casting use the same mold, the mold constant B would be equal to each other. When applying the 25 % difference, the equation can be simplified to:

(2) $${\begin{array}{c} (\frac{V}{A})\; \end{array}}_{Riser}^{2}=1.25\;{(\begin{array}{c} \frac{V}{A} \end{array})\;}_{Casting}^{2}$$

The riser has a cylinder shape with a volume of 10.75 cm^3^ and a surface area of 33.13 cm^2^. Following Eq. (2), the riser had a solidification time 26 % slower than that of the casting. Because the mold and the casting both had to cool down from the same temperature when removed from the furnace, the riser and casting solidification process started once the mold walls had cooled down to the solidification temperature. Assuming that every part of the mold wall cools down equally, the shrinkage cavities start occurring in the middle. Therefore, the riser was positioned above the middle part of the casting so gravity forces the melting material into the casting in an efficient way (Figure 3). The setup was used to cast two ingots at 420 °C, that were machined into dog-bones and 2 cylinders. The cylinders with a diameter between 30 and 50 mm were used for material analysis. The dog-bone samples were designed in accordance with the ASTM E8 standard [30] and were used to carry out the tensile tests (Supplemental file 4). Furthermore, an ingot made from virgin Zamak was made and used to machine the dog-bones.

### 2.2 Material validation experiments

To determine the purity of all of the cast ingots, they were compared with samples made from virgin Zamak 3. In order to determine the influence of the coating also ingots were cast from laryngoscope blades from which the coating was removed by means of a chemical dissolving agent (Superafbijt 507645, Pearl Paint Holland BV, Lelystad – the Netherlands).

All of the cylindrical samples are analyzed using X-ray fluorescence (XRF) spectroscopy (Panalytical Axios Max WD-XRF, Malvern, United Kingdom). The purity is calculated with SuperQ5.0i/Omnian software to define the relative proportions of zinc, aluminum, magnesium and copper.

The dog-bones and tested material properties were used to determine the stress-strain curves. The Ultimate Tensile Strength (UTS) was defined as the highest measured tensile strength before breakage. The Yield Strength (YS) is defined as the first point in the graph the stress remains constant during elongation. The Young's Modulus is defined in this study as YS/strain.

### 2.3 Part manufacturing, a test case

**Figure 4:**
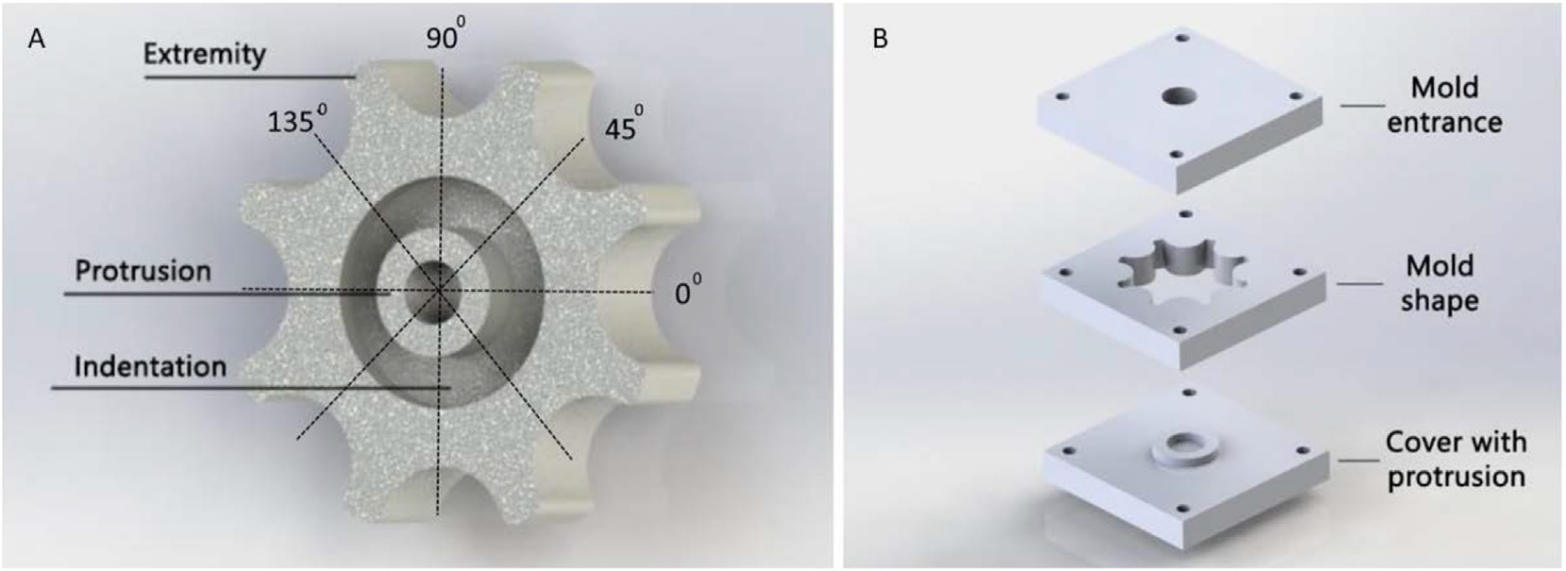
Control wheel casting. A: Left, the simplified cast. B: Right, mold design.

To investigate whether the “all-in-one” process can be used to manufacture functional quality parts, a stainless steel casting mold for a control wheel is made. The control wheel is part of the handle of the SATA instrument, a medical instrument used for laparoscopy [31]. This specific wheel had a complex shape with multiple cutouts and an inner chamber making this medical part the ideal test subject. The mold shown in Figure 4, consists of 3 slabs, each 10 × 55 × 55 mm in size, held to together by nuts and bolts. The wheel ingot was cast with the same melting setup after heating the laryngoscope blades for an hour on 420 °C. To determine the level of shrinkage, the most relevant control wheel dimensions were measured and compared to the dimensions of the original shape. A T-test (equal variance, two tailed) was conducted to see if differences were found between the component and cast dimensions. A probability of p < 0.05 was considered to be statistically significant. Measurements were conducted with a Mitutoyo digimatic caliper. The dimensions of the four extremities shown in Figure 4 were measured in an alternating way. Protrusion and indentation were measured on four locations on 0, 45, 90 and 135° (Figure 4-Left). The shrinkage was determined by calculating the percentage the control wheel dimensions deviated from the cast shape dimensions.

The edges of the inner shape of the control wheel cast were not rounded to investigate the potential of the Zamak to form uniform sharp edges within the process. An electric microscope system with zoom capability up to 10,000× (MF measuring microscope, Mitutoyo, Kanagawa, Japan) was used to determine the roughness of the edges on four locations on a pair of opposing extremities in order to quality assure the cast parts. A T-test (equal variance, two tailed) was conducted to see if differences were found between the component and cast dimensions. a probability of p < 0.05 was considered to be statistically significant. Finally the wheel surfaced was machined to match the exact properties of a functional wheel by machining the rims and surfaces on a lathe. A hole was drilled and a thread cut inside the hole for a fixation screw. The wheel was placed on an instrument for functional testing of the thread during and after assembly and the thread was optically inspected after disassembly.

## 3 Results

**Figure 5:**
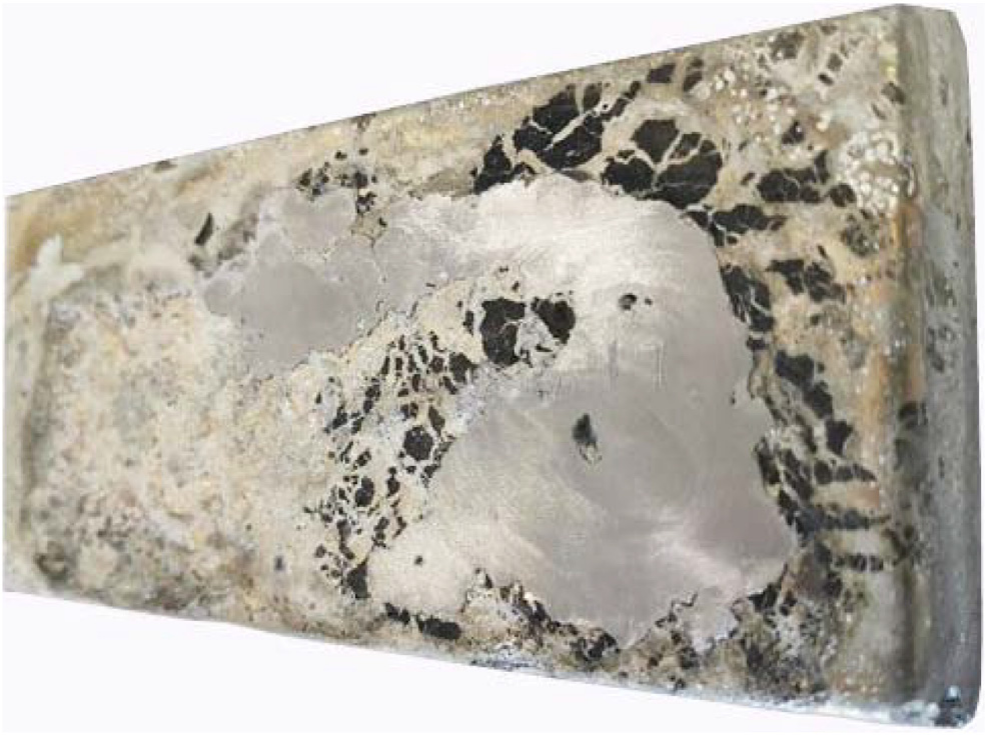
Ingot after ultrasonic cleaning.

Two batches of laryngoscope blades with a total mass of 48 kg were successfully collected and separated from the hospitals and melted and cast. An example of a produced ingot is given in Figure 5.

The amount of Zamak extracted from the blades per melting setup is provided in Table 2. The melting setup resulted in approximately 93% recovered Zamak over a time span of 3 h.

**Table 2:** Results of the weighing test for the melting setup.

Temp	Number of blades used	Zamak mass before casting	Zamak mass after casting	Zamak recovery %
420 °C	10	750 g	680 g	91.9 %
420 °C	10	742 g	700 g	94.4%

The results from the XRF tests show that although impurities were present in the castings, they mainly contained Zinc. The tested ingots S2-C and S2-D had a purity of 99.7 % (Table 3). The full material composition data from the XRF tests can be found in Supplemental file 3.

**Table 3:** Chemical composition of the cast ingots.

Element Concentration	Ingot A	Ingot B	Coating removed	Virgin Zamak
Zinc (Zn)	94.97%	95.65%	96.36%	91.55%
Aluminum (Al)	4.28%	3.78%	3.3%	4.5%
Magnesium (Mg)	0.42%	0.25%	0.17%	0.8%
Copper (Cu)	0.04%	0.04%	0.02%	2.8%
Iron (Fe)	0.04%	0.05%	0.01%	0.01%
Nickel (Ni)	0.01%	0.02%	-	0.01%
Silicon (Si)	0.12%	0.1%	0.05%	0.05%
Chloride (Cl)	0.08%	0.04%	0.17%	0.17%
Sulfur (S)	0.03%	0.02%	0.04%	0.01%
Phosphorus (P)	0.01%	0.01%	-	0.01%
Potassium (K)	-	0.01%	0.04%	0.03%
Calcium (Ca)	-	0.02%	0.02%	0.02%
Fluorine (F)	-	-	-	-
Chromium (Cr)	-	0.02%	-	-
Total Purity %	99.7%	99.7%	99.85%	99.7%

**Figure 6:**
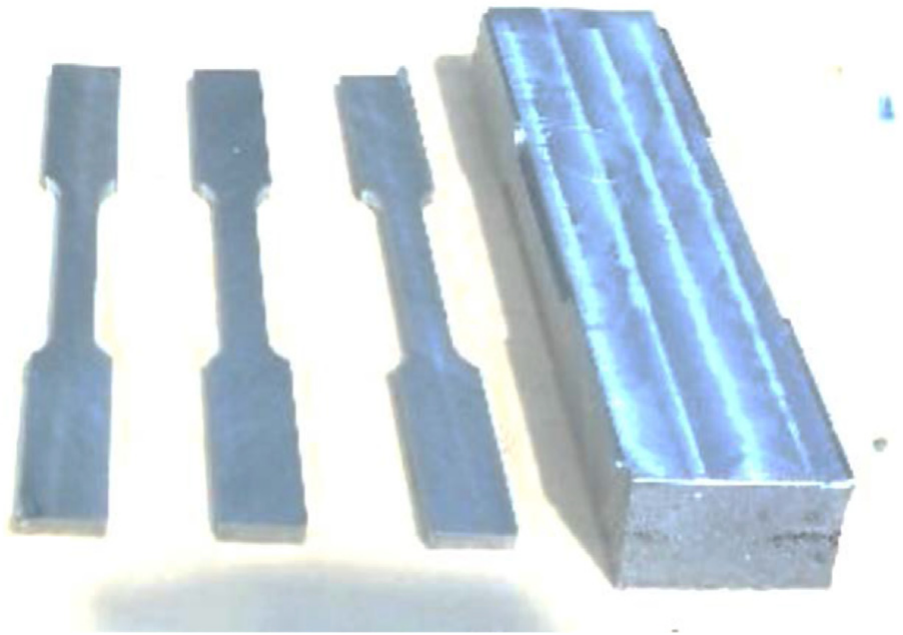
Dog-bone samples machined from the cast ingots.

Examples of machined dog-bone samples for tensile testing, according to ASTM E8, are shown in Figure 6.

### 3.1 Mechanical properties

**Figure 7:**
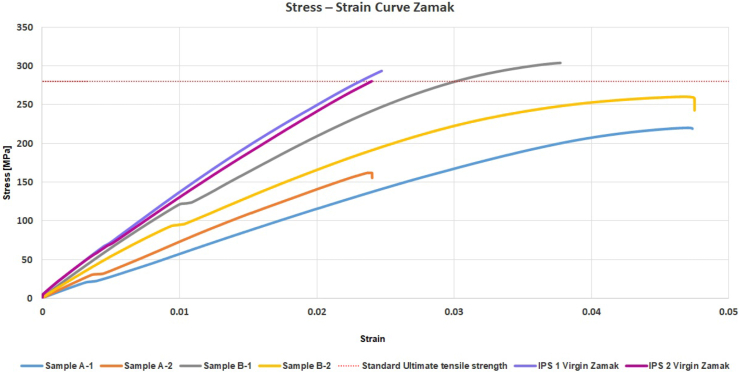
Stress-strain curve of the tensile tests with dog-bones.

The results of the tensile tests are shown in Table 4. Figure 7 shows the stress-strain curves of two dog-bones from each cylinders (A1, A2, B1, B2), and two virgin dog-bones (IPS 1, IPS2). The individual stress-strain curves of each sample are shown in Supplemental file 4. The average UTS of reprocessed Zamak was 236 ± 61 (MPa), being 82% of the averaged UTS of standard Zamak (287 MPa). The average YS of the reprocessed Zamak was 103 % or 70 ± 43 MPa when comparing to standard (virgin) Zamak (68 MPa). The average Young's Modulus of the reprocessed Zamak was 64 % or 9 ± 3 GPa of the standard Zamak (14 Gpa).

**Table 4:** Results of the tensile tests.

Zamak Properties Tensile Test	Ultimate Tensile Strength [MPa]	Yield Strength [MPa]	Young's Modulus [GPa]
***Standard Properties Literature***	280 MPa	*210*	*86*
**Ingot Virgin IPS**			
*Sample IPS-1*	293	67	*14,6*
*Sample IPS-2*	280	68	*14,3*
**Ingot A**			
*Sample A-1*	220	43	6.8
*Sample A-2*	161	28	8,3
**Ingot B**			
*Sample B-1*	304	124	12,3
*Sample B-2*	260	86	10
**Ingot** average, SD/±	236 ± 61	70 ± 43	9 ± 3

### 3.2 Part manufacturing, a test case

**Figure 8:**
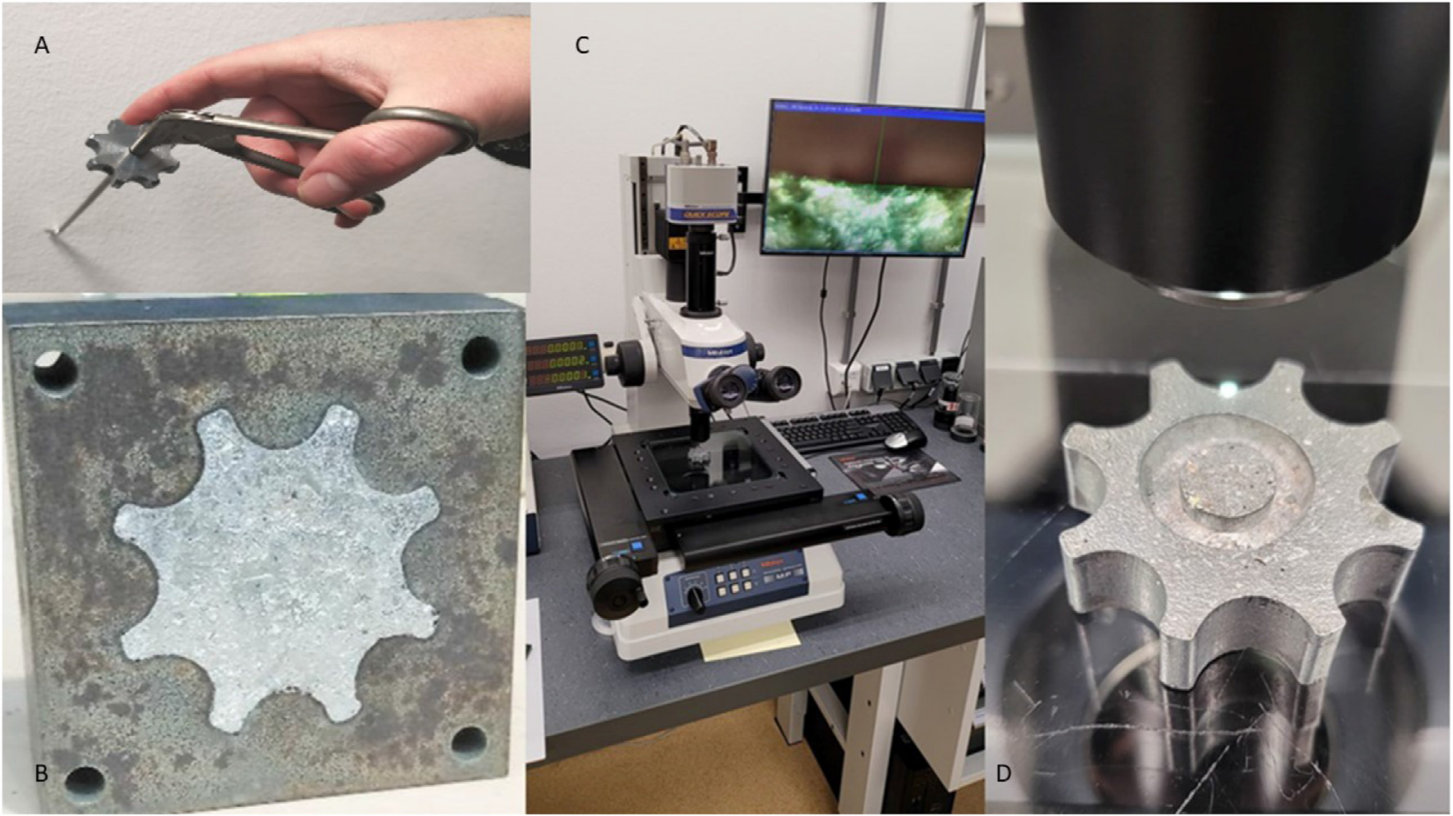
Assessment of the final part. Top left. A: Finalized control wheel fixed on the instrument to test the fixation strength. Below Left. B: A very tight fit established between the component and cast half fabricate. C & D: Middle and Right, measuring microscope with cast control wheel close up.

Casting turned successful (Figure 8) and only three undesired surface faults were detected with a maximal depth of 0.6 mm along the edges of the protrusion. It was possible to remove the component from the cast within 10 min by pressing the wheel from its cast with a 0.5 tonnage rack and pinion press. The casts did not show any damage after removal. Table 5 shows the measured dimensions of both cast and control wheel.

**Table 5:** Pattern shrink percentages of different sections of the rotation wheel (averaged over 4 measurements).

Description	Outer diameter wheel [mm]	Inner diameter wheel [mm]	Outer diameter cutout [mm]	Inner diameter cutout [mm]	Thickness rims [mm]	With rims [mm]	Height cutout [mm]	Sharpness corner rim 1 [mm]	Sharpness corner rim 2 [mm]
**Location**									
**Product measurements**	17,95	9,9	37,96	27,08	10,22	3,62	3,03	0,02	0,02
	17,8	9,89	37,92	27,03	10,24	3,6	3,09	0,02	0,05
	17,84	9,87	37,94	27,07	10,19	3,58	3,07	0,04	0,03
	17,79	9,9	37,92	26,98	10,23	3,62	3,02	0,04	0,06
***Mean***	***17,845***	***9,89***	***37,935***	***27,04***	***10,22***	***3,605***	***3,0525***	***0,03***	***0,04***
***SD***	***0,07***	***0,01***	***0,02***	***0,05***	***0,02***	***0,02***	***0,03***	***0,01***	***0,02***
	17,96	9,89	37,98	27,03	10,19	3,73	3,12	0,02	0,04
	17,92	9,9	38,02	27,23	10,25	3,61	3,08	0,03	0,03
	17,97	9,92	37,91	27	10,23	3,62	3,09	0,03	0,04
	17,99	9,89	38,24	27,01	10,21	3,63	3,07	0,04	0,03
***Mean***	***17,96***	***9,9***	***38,0375***	***27,0675***	***10,22***	***3,6475***	***3,09***	***0,03***	***0,04***
***SD***	***0,03***	***0,01***	***0,14***	***0,11***	***0,03***	***0,06***	***0,02***	***0,01***	***0,01***
**averaged difference**	**0,12**	**0,01**	**0,10**	**0,03**	**0,00**	**0,04**	**0,04**	**0,00**	**-0,01**
**Difference in % (shrinkage)**	**0,64**	**0,10**	**0,27**	**0,10**	**0,00**	**1,17**	**1,21**		
**P value (t-test)**	**0,06**	**0,53**	**0,28**	**0,68**	**1,00**	**0,17**	**0,18**		

The shrink varied between 0.0 % and 1.2 % and varied over the individual extremities. The shrink for the thickness of the control wheel was around 0.0 %, whereas the shrink for the height of the cutout was around 1.2 %. The sharpness of the corners was measured to be around 0.01 mm.

## 4 Discussion

### 4.1 Mechanical properties

The results of this study show that melting Zamak disposable blades into raw material to make new parts and components is feasible. Melting at 420 °C for 3 h resulted in extraction of 93% of laryngoscope blade Zamak. The tensile tests revealed that the mechanical properties of the reprocessed laryngoscope blades were lower compared to the standard Zamak properties from the literature. Nevertheless, they were in line with the tested virgin Zamak (IPS). The averaged UTS, yield strength and Young's modulus were 82 %, 103 % and 64 % of virgin Zamak (IPS). Within our “all-in-one” processing method, gravity casting was used. The most noticeable results of the mechanical tests were the values of the YS of all tested samples in relation to the theoretical YS of 280 MPa. In all cases, the values remained much lower, indicating a significant difference in the location of the yield point. In case of pure Zamak, the stress strain curve was more linear compared to the tested samples. Therefore, it is likely that the theoretical YS is based on the 0.2 % offset method [32] instead of using the true Yield point. Another factor which may have affected the mechanical qualities were the impurities [33]. Although it is likely that most observed impurities on the surface of the ingots came from the coating and the other plastic parts of the laryngoscope blades, the XRF data showed that removal of the coating did not result in significant differences in composition regarding impurities. The amount of Magnesium and Copper, appeared to be much higher for Virgin Zamak. The amounts for Magnesium appeared to be more than twice as high for Virgin Zamak compared to the samples made from the blades. The amounts of Copper appeared to be more than 50 times as high compared to the samples made from the blades. Copper increases the strength of Zamak however, as an alloy it also has a relative higher cost price. For casting laryngoscope blades in high volumes, the cost prices as well as the influence on the elongation at break can be a determining factor as to why the blades contain less copper. The decrease in UTS might be influenced by the decrease of copper and magnesium in the ingots when compared to the virgin Zamak as these alloys contribute to a higher structural strength and corrosion resistance. More testing is needed to determine the cause for the very wide YS range before it is possible to set up a proper Quality Control System when components are made.

The Yield strength showed a relative low outcome with ingot A which might have been influence by a potential cavity in the dog-bone or a flaw defect during clamping on the tensile bench. The aim of this study however, was to validate the process with a gravity cast component to be used to steer a tip of an instrument. The application of using the control wheel takes place with relative low force and well below the yield strength. Additional study is needed however, to eliminate potential flaws such as cavities or cracks in the dog-bones or slippage during tensile testing. The mechanical properties with regard to the loading upon the end product, the steering wheel, is below the mean YS and UTS during operation, washing, disinfection, sterilization and transport in a hospital environment. The instrument is operated with thumb and ring finger. The index finger turns the steering wheel. The steering wheel causes the tip of the instrument to rotate. The forces exerted on it with the fingers are very limited. These will have no effect on plastic deformation of the material. Interesting would additionally be to further investigate the density of the material after gravity casting of the control wheel.

Follow up studies should furthermore, indicate if the Zamak, extracted from the process is free from DNA or RNA traces. Also should this study be further expanded in order to retrieve more test data to gain more statistical insights.

### 4.2 Ingot porosities

The concentrations of Zinc and silicon found in the ingots after casting were higher than in virgin Zamak. The silicon is likely coming from the plastic parts of the laryngoscope blades. No significant porosities were observed in the ingots. Therefore, it is likely that gas porosity is prevented due to the use of the Zamak recommended casting temperature of 420 °C and the presence of a riser that prevented shrinkage porosity. This is supported by the lack of visual signs of shrinkage porosity. The observed contaminants and inclusions most likely came from entrainment of liquid/gas that was sucked out of the surrounding material as the pressure in the flowing medium was lower compared to its environment [34].

### 4.3 Gravity casting process

The results showed that it is feasible to create an “all-in-one” Zamak casting process for functional new parts with a minimal production setup as alternative for die-casting. Although it demonstrated to be possible to incorporate specific details into the stainless cast-design without significant shrinkage, some minor flaws were detected along the edges of the wheel part. Therefore, it is suggested to investigate if the inflow of material in the indentations and protrusions of the cast can be improved by adjusting the height of the setup or by controlling the cooling down of the setup. The used molds were manufactured from 10 mm thick stainless steel plate with multiple sections. It is likely that the segments with differing surface areas and volumes have different cooling rates. As this can lead to non-uniform shrinkage as observed in de indentation cut-out, the segments in the mold design should compensate for this by removal or adding material around the wall of the mold to create a more uniform wall thickness. A more uniform wall thickness and controlled cooling can result in less material tension in the component leading to better tolerances. To investigate the influence of the relative position in the setup on the melt composition, it is interesting to determine the melt composition quality for multiple material flow locations in the “all-in-one-system. Finally, it is advised to look at larger scale Zamak reprocessing initiatives weather learned lessons can be incorporated in our product design and Zamak melting and casting processes.

Our study focused on the technical recycling of medical materials such as Zamak. An interesting topic would be to investigate the use of biodegradable materials as source of raw material for laryngoscope blades. Biodegradable materials break down naturally for which no chemicals are needed. The introduction of biodegradable materials as source for medical devices as well as certification of these materials under CE MDR requires further exploration.

### 4.4 Circular economy

Despite the observed limitations, the results indicated that is feasible to use medical waste, such as laryngoscope blades, as source of raw material and semi-finished components. After this process is further matured and scaled, it can contribute to reduce medical waste. Reducing waste within the Circular Economy is growing in significance due to its benefits to society [35, 36, 37]. Reusing and reprocessing disposable medical devices after sterilization have been reported earlier [38]. The reuse of Zamak laryngoscope disposables in their current design may significantly contribute to CO_2_ reductions but is legally restricted [39]. Melting, as a second option, will require more energy than (re)sterilizing but is more sustainable than incineration. Before environmental claims can be made about the benefits of using disposed Zamak as input material for new products, a Life Cycle Assessment (LCA) study needs to be conducted on the “all-in-one” process versus the standard in order to calculate environmental impacts categories such as water- and land use, fine particulate matter and eco-toxicity. To investigate the costs of reprocessing versus the manufacturing of components made out of virgin and reprocessed Zamak, an in-depth cost study, such as a Life Cycle Costing (LCC) should be conducted.

### 4.5 Study limitations

Although not included in the scope of this study, a cost analysis of the logistical process of the urban mined laryngoscope blades, melting and casting would be an area of interest for further investigation as this has not been explored in this study. Special logistical set-up was needed, as these blades have been used with patients in the Operating Room, meaning that they are potentially contaminated, generating more costs. These costs include safe-handling of the waste, use of validated and special containers with closable lids and the costs of the process of thermo-disinfection. These costs should be compared with the standard current costs for hospitals when disposing medical waste. With regard to the casting procedure, additional research is needed to determine whether the Zamak quality remains constant with gravity casting as compared to die-casting. Furthermore, the material use and quality consistency of molds, used during the casting, is an area to be explored additionally. Although both blades as test samples were made from Zamak 3, we are not 100% sure that the material was made according to exactly the same formula and composition. The influence of changes in the composition that are still within the boundaries of Zamak 3, the material properties should be further investigated. When parts for medical devices are cast in a later phase of process development, density testing, clinical testing and evaluation is required.

## 5 Conclusion

An “all-in-one” process was designed using disposed Zamak laryngoscope blades that were successfully reprocessed into raw material and directly molded into a surgical instrument component. After being optimized, a gravity driven “all-in-one” reprocessing setup for Zamak disposables can be an affordable alternative for expensive die casting methods. Despite the changing mechanical properties as compared to standard Zamak, reprocessing Zamak laryngoscope blades demonstrated to have potential for making new (medical) parts. The cast Zamak parts still require post-processing, and is not yet an ‘as-ready’ product when the part was released from the mold. However, this study demonstrated the feasibility of reprocessing Zamak medical waste into new components contributing to the development of a circular health care economy.

## Declaration

### Author contribution statement

Bart van Straten, PhD, MBA; Tim Horeman, PhD: Conceived and designed the experiments; Performed the experiments; Analyzed and interpreted the data; Contributed reagents, materials, analysis tools or data; Wrote the paper.

Brian Tantuo, Msc: Conceived and designed the experiments; Performed the experiments; Analyzed and interpreted the data; Wrote the paper.

Jenny Dankelman, PhD: Performed the experiments; Analyzed and interpreted the data; Contributed reagents, materials, analysis tools or data; Wrote the paper.

Nicolaas H. Sperna Weiland, PhD; Bendiks-Jan Boersma, PhD: Performed the experiments; Contributed reagents, materials, analysis tools or data.

### Funding statement

This research did not receive any specific grant from funding agencies in the public, commercial, or not-for-profit sectors.

### Data availability statement

Data included in article/supp. material/referenced in article.

### Declaration of interest’s statement

The authors declare no conflict of interest.

### Additional information

No additional information is available for this paper.

## Appendix A Supplementary data

Supplemental file 1 090922.docx Supplemental file 1 090922.docx

Supplemental file 2 020122.docx Supplemental file 2 020122.docx

Supplemental file 3 020122.docx Supplemental file 3 020122.docx

Supplemental file 4 020122.docx Supplemental file 4 020122.docx
