# Supplementary material for: Reprocessing Zamak laryngoscope blades into new instrument parts; an ‘all-in-one’ experimental study
Source: Heliyon. 2022 Nov 17;8(11):e11711. doi: 10.1016/j.heliyon.2022.e11711 (PMC9679383; doi:10.1016/j.heliyon.2022.e11711)
Supplement: Supplemental file 1 090922.docx [file mmc1.docx]

**Supplemental file 1: “All-in-one” process.**

A process diagram of the instrument flows during reprocessing is shown in Figure 1.

*
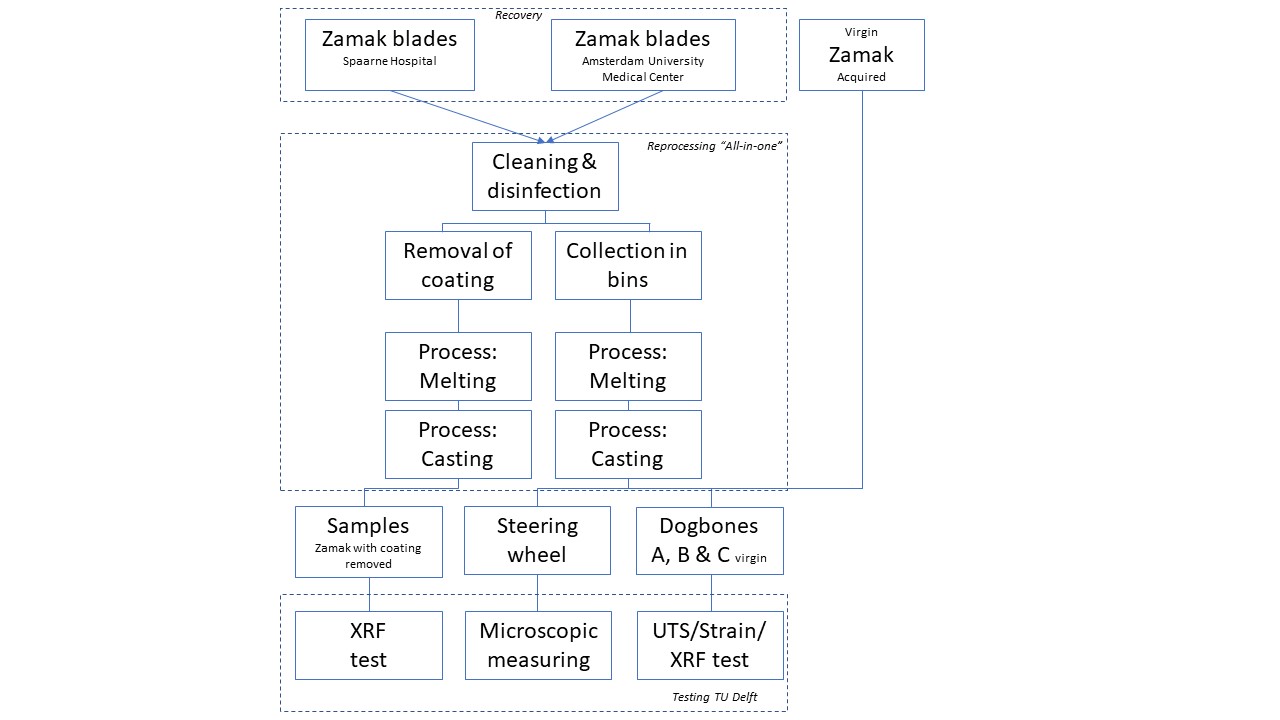
*

Figure 1. Process diagram of instrument flows.

The extraction, casting and manufacturing of Zamak will occur in a single system without the user needing to continuously interact with it. By applying this concept to the laryngoscope blades, the recovery of Zamak and manufacturing of new products can sequentially be achieved. An “All-in-one” process for melting of Zamak in conjunction with casting it without additional manual steps in between is shown in Figure 2.

**
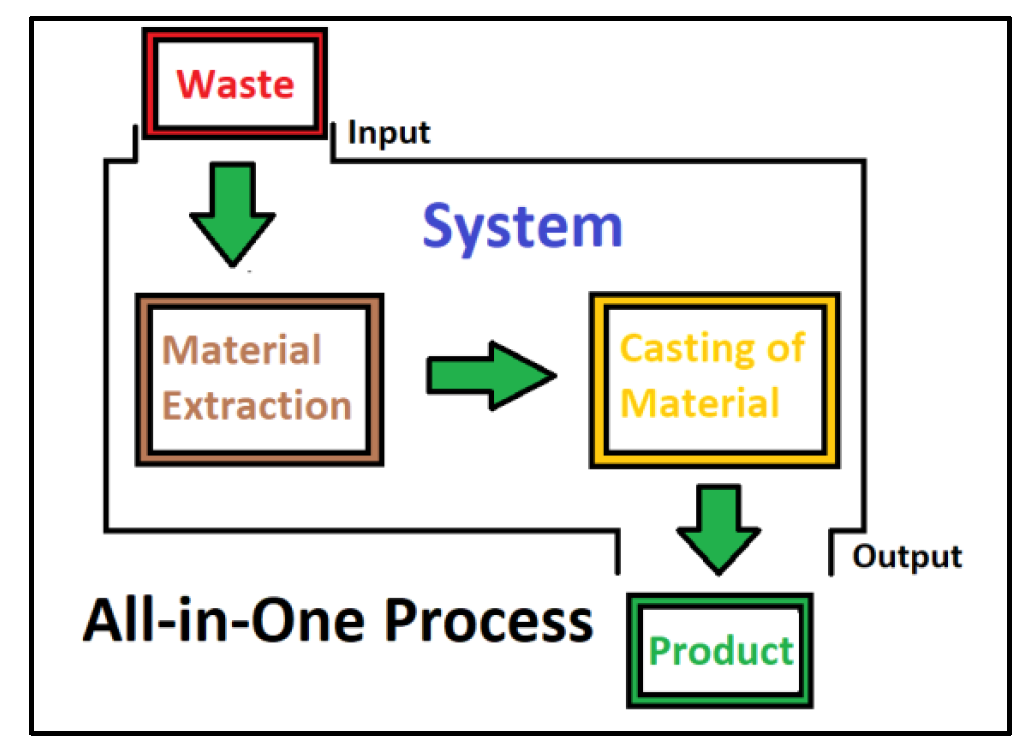
**Figure 2. Schematic of the “all-in-one” process with recovered laryngoscope blades, melting and casting of new products in a single production line.

The melting, in an electric melting oven, KOS, series 219029, as well as the casting is done in a single production line, based on one location. Disinfection, melting, casting in one production line as shown in Figure 3.


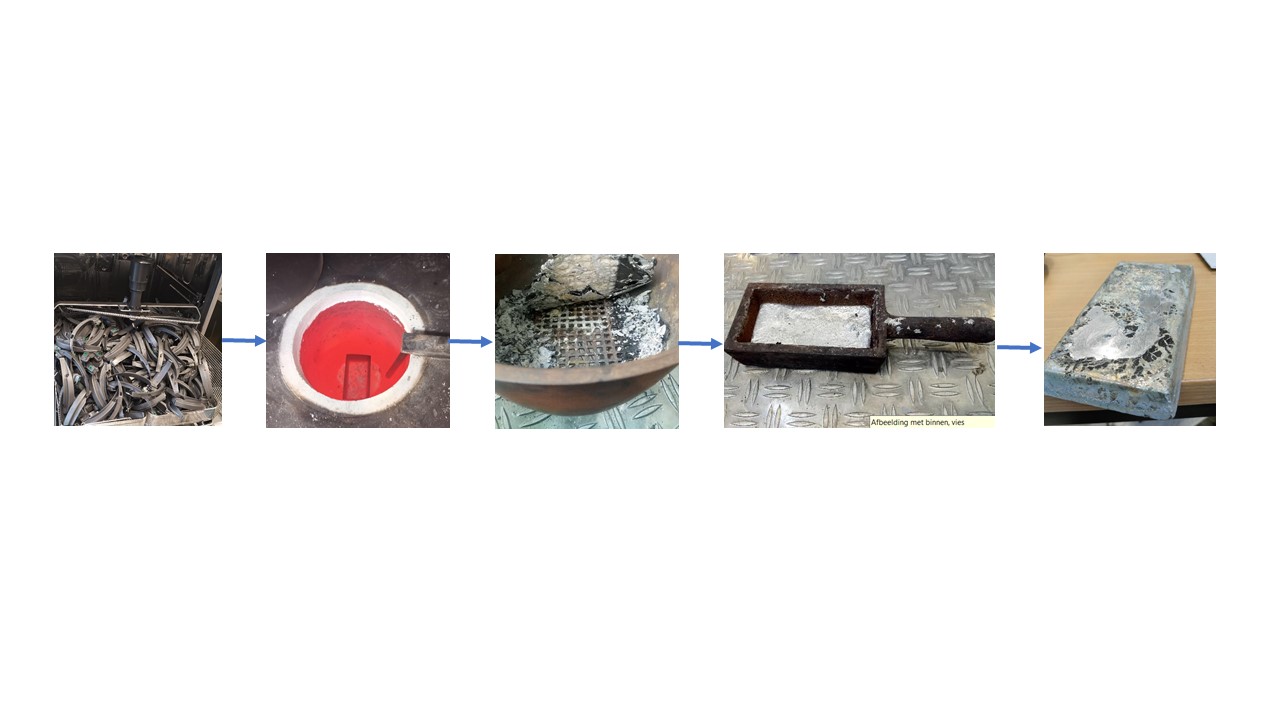


Figure 3. “All in One” process on one location.
