# Supplementary material for: Reprocessing Zamak laryngoscope blades into new instrument parts; an ‘all-in-one’ experimental study
Source: Heliyon. 2022 Nov 17;8(11):e11711. doi: 10.1016/j.heliyon.2022.e11711 (PMC9679383; doi:10.1016/j.heliyon.2022.e11711)
Supplement: Supplemental file 2 020122.docx [file mmc2.docx]

**Supplemental file 2: XRF spectroscopy test results**

**Ingot A* Ingot B***

**
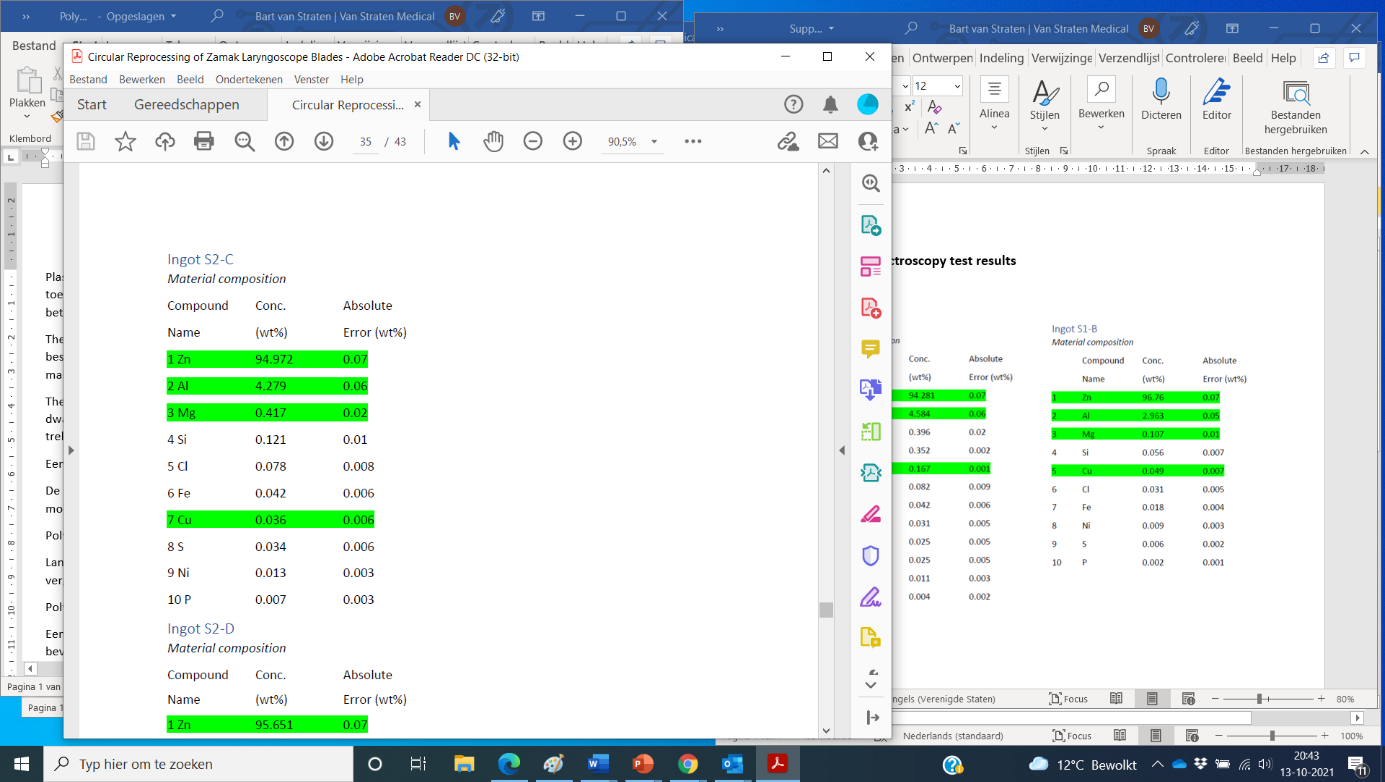

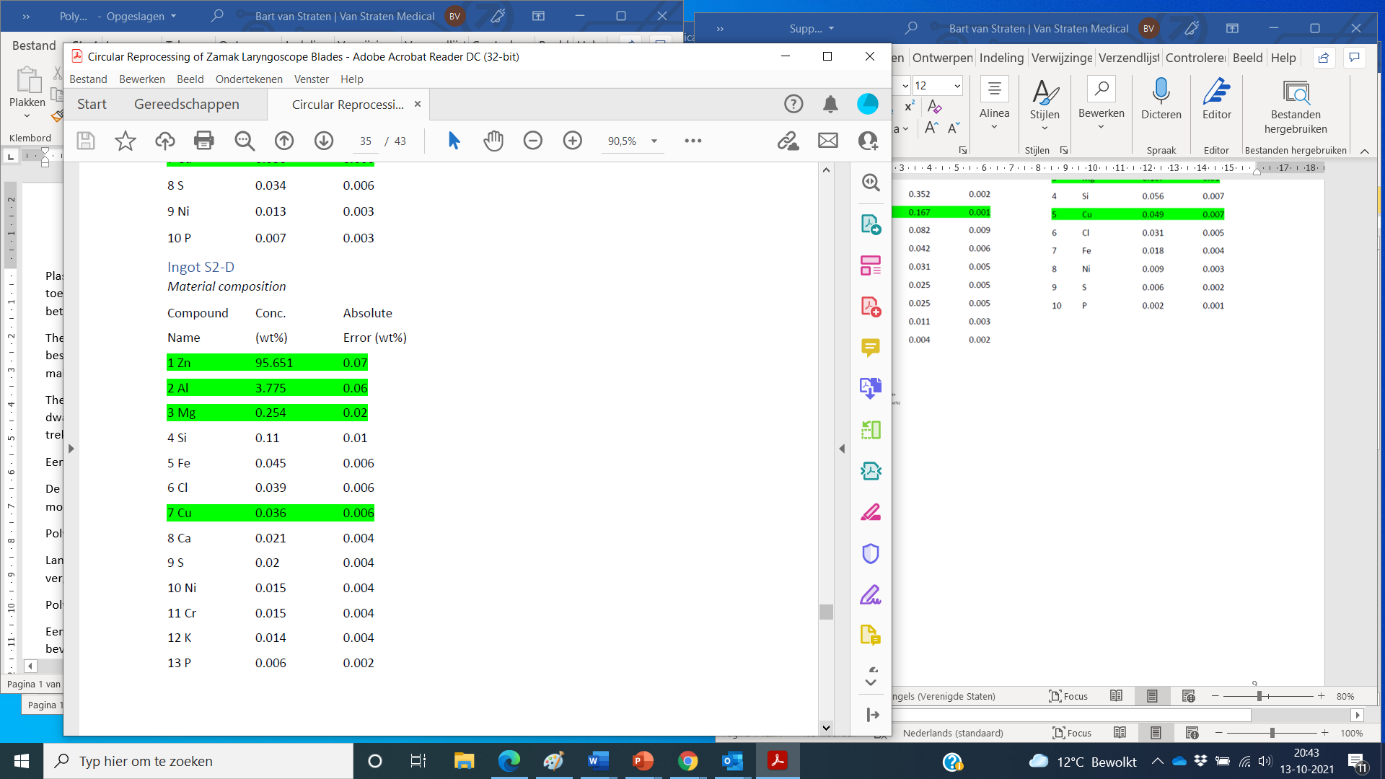
**


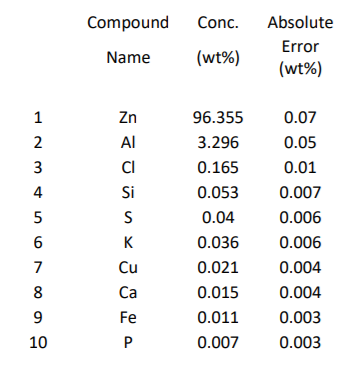
**Zamak with coating removed before melting* Virgin Zamak***


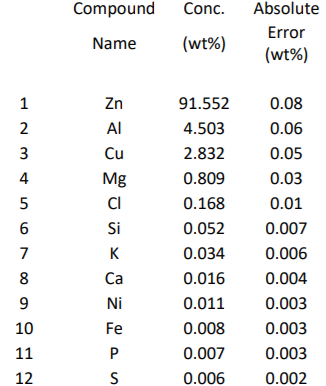


* Ruud Hendrikx at the Department of Materials Science and Engineering of the Delft University of Technology is acknowledged for the X-ray analysis.
