# Supplementary material for: Reprocessing Zamak laryngoscope blades into new instrument parts; an ‘all-in-one’ experimental study
Source: Heliyon. 2022 Nov 17;8(11):e11711. doi: 10.1016/j.heliyon.2022.e11711 (PMC9679383; doi:10.1016/j.heliyon.2022.e11711)
Supplement: Supplemental file 3 020122.docx [file mmc3.docx]

**Supplemental file 3: Dog-bones**

Dog bone samples were used to carry out the tests. Figure 1 shows the size and dimensions. The dog bone samples were designed in accordance with the ASTM E8 standard [1]. They were created by first milling the Zamak ingots according to the designed dog bone and afterwards sawing them in slices.


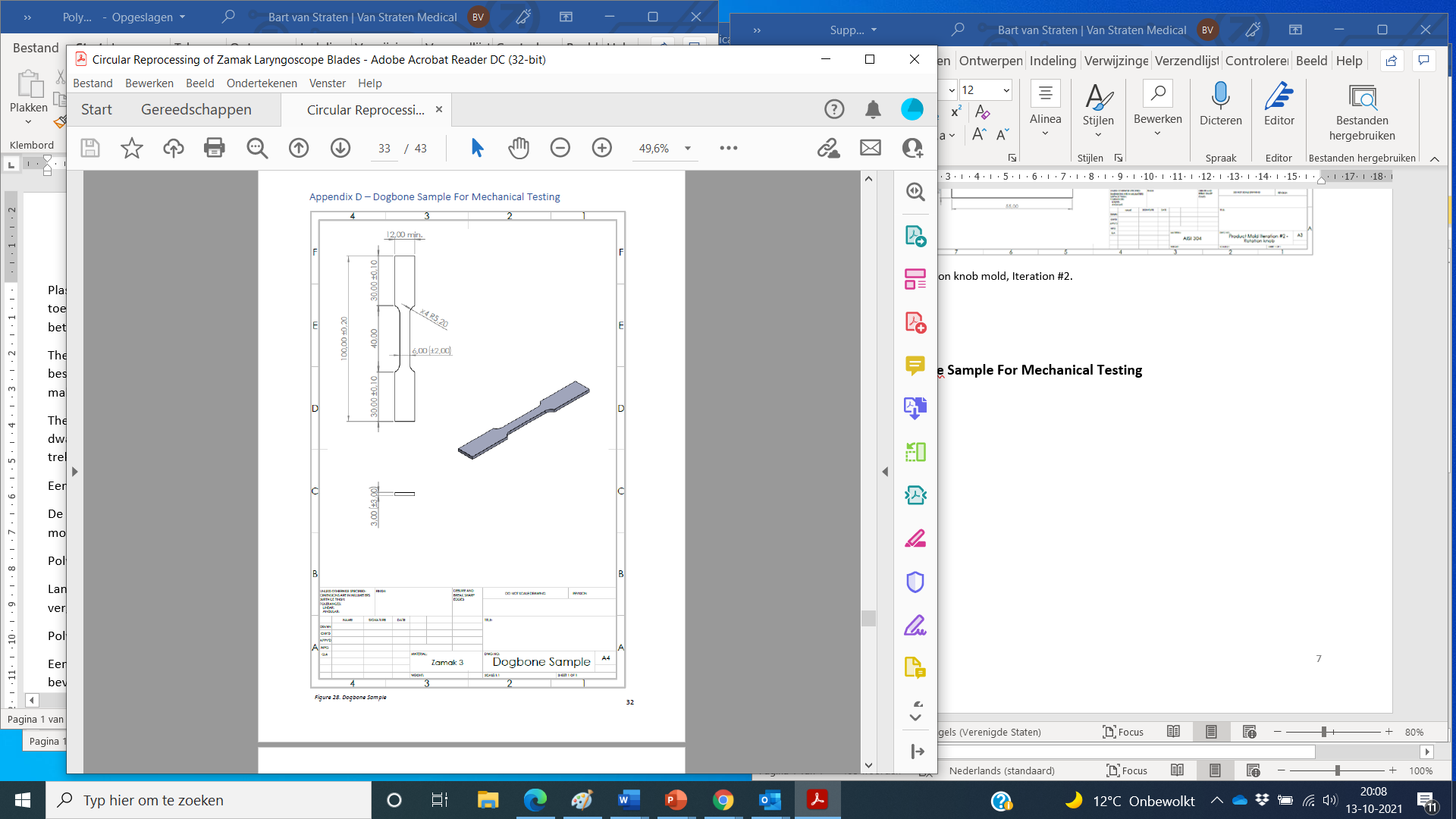


Figure 1. Dog bone Sample

References

1. Davis, J. R. (Ed.). (2004). Tensile testing. ASM international. p. 52
