# Supplementary material for: Reprocessing Zamak laryngoscope blades into new instrument parts; an ‘all-in-one’ experimental study
Source: Heliyon. 2022 Nov 17;8(11):e11711. doi: 10.1016/j.heliyon.2022.e11711 (PMC9679383; doi:10.1016/j.heliyon.2022.e11711)
Supplement: Supplemental file 4 020122.docx [file mmc4.docx]

**Supplemental file 4: Stress-strain**

Samples were made from Ingots A (A1 and A2) and B (B1 and B2) and from virgin Zamak-3 (IPS1 and IPS2). This supplemental file shows the combined stress-strain relationships as well as each individual curve per sample.


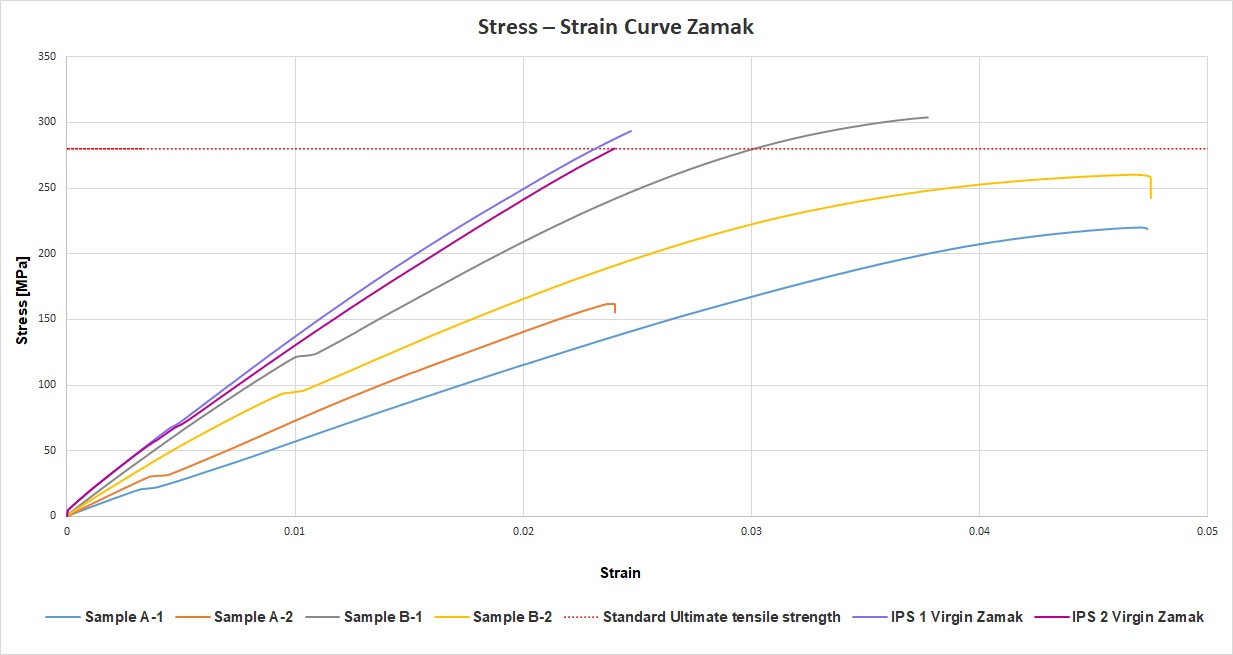


|  |  | UTS | YP | Strain |
| --- | --- | --- | --- | --- |
|  | A1 | 220 | 20.25 | 4.70% |
|  | A2 | 161.63 | 29.96 | 2.40% |
|  | B1 | 303.87 | 121.8 | 3.77% |
|  | B2 | 258 | 93.8 | 4.70% |
|  | IPS 1 V | 293.22 | 67 | 2.50% |
|  | IPS 2 V | 280.2 | 67 | 2.40% |
|  |  |  |  |  |
|  |  | Strain @ YP | YM |  |
|  | A1 | 0.3 | 6.75 |  |
|  | A2 | 0.36 | 8.322222 |  |
|  | B1 | 0.99 | 12.30303 |  |
|  | B2 | 0.94 | 9.978723 |  |
|  | IPS 1 V | 0.46 | 14.56522 |  |
|  | IPS 2 V | 0.47 | 14.25532 |  |
